# Supplementary material for: Interference control in working memory: Evidence for discriminant validity between removal and inhibition tasks
Source: PLoS One. 2020 Dec 2;15(12):e0243053. doi: 10.1371/journal.pone.0243053 (PMC7710115; doi:10.1371/journal.pone.0243053)
Supplement: S1 Table — (PDF) [file pone.0243053.s001.pdf]

Table S1

Bayes Factors in favor of the Alternative Hypothesis (BF_{10}) and in favor of the Null hypothesis (BF_{01}) for the Pearson Correlation Coefficients.

	BF	Number Stroop	Arrow flanker	Local	Simon	Neg. comp.	Antisaccade	Letter upd.	Digit upd.
Arrow flanker	BF_{10}	**0.10**							
	BF_{01}	*9.74*							
Local	BF_{10}	**0.13**	**0.09**						
	BF_{01}	*7.80*	*11.62*						
Simon	BF_{10}	**0.08**	**0.08**	**0.33**					
	BF_{01}	*13.22*	*12.22*	*3.02*					
Neg. comp.	BF_{10}	**0.08**	**0.08**	**0.11**	**0.08**				
	BF_{01}	*12.46*	*12.95*	*9.21*	*12.42*				
Antisaccade	BF_{10}	**0.11**	**0.10**	**0.08**	**0.09**	**0.24**			
	BF_{01}	*9.25*	*10.45*	*13.24*	*11.39*	*4.09*			
Letter upd.	BF_{10}	**0.26**	**0.09**	**1.08**	**0.19**	**0.08**	**0.13**		
	BF_{01}	*3.79*	*11.05*	*0.92*	*5.13*	*13.24*	*7.48*		
Digit upd.	BF_{10}	**0.30**	**0.08**	**0.14**	**0.08**	**0.17**	**0.08**	**939.95**	
	BF_{01}	*3.37*	*12.44*	*7.19*	*12.68*	*5.79*	*12.18*	1.06×10^{-3}	
Word upd.	BF_{10}	**0.17**	**0.14**	**0.43**	**0.08**	**0.08**	**0.09**	**431.89**	$\mathbf{3.24 \times 10^{3}}$
	BF_{01}	*5.86*	*7.40*	*2.34*	*12.71*	*12.64*	*11.31*	2.32×10^{-3}	3.09×10^{-4}

Note. For the sake of clarity, BF_{10} are presented in bold, whereas BF_{01} are presented in italics. The BF were estimated in R [60] using the BayesMed package [68] with default prior scales. BF = Bayes Factor; Neg. comp. = Negative compatibility; upd. = updating.
