# Supplementary material for: Interference control in working memory: Evidence for discriminant validity between removal and inhibition tasks
Source: PLoS One. 2020 Dec 2;15(12):e0243053. doi: 10.1371/journal.pone.0243053 (PMC7710115; doi:10.1371/journal.pone.0243053)
Supplement: Attachment — Submitted filename: Response To Reviewers.docx [file pone.0243053.s002.docx]

# Editor

***E1 “As you will see, the reviews are mixed. In particular, one of the Reviewer is satisfied with the changes you have made. The other one still see some substantial -theoretical- weakness in your study. I have also looked at the revised version of your interesting paper. I have to say that I agree with the comments done by Reviewer 1 . I am thus asking you to revise the paper carefully taking into consideration the points made by Reviewer 1 that mainly regards the introduction and the discussion section. The points the Reviewer raised are important from a theoretical point of view, and by considering and "including" them in your revision (which means also clarifying some debated aspects on the construct you are working in), your paper will have a strong impact in the literature on inhibition.”***

RESPONSE: We revised the manuscript carefully, in particular the introduction and the discussion, and we clarified the formulation regarding the concept of inhibition where appropriate (see p. 2-3, 5-7, 20, 24, 28, 30-31, 33-34, and 37-38). Nevertheless, we still use the descriptive terms “inhibition task” or “inhibition factor” in the manuscript. Given their use is convention, we think that this allows readers to readily grasp what task or factor we are referring to.

# Reviewer A

## *A1 “To be honest, I thought my comments to the previous version of the manuscript would prompt the authors to somehow refocus the introduction/discussion (they seem to agree with me in relevant theoretical points according to their responses on the letter to reviewers). This has not been the case, however, with the revised manuscript only including a new few lines in the discussion. Well, this is their manuscript, not mine. However, I do not think the manuscript has been significantly improved. If the authors (like me) believe that their ‘inhibition’ tasks mostly tap into (mechanism-free) interference control (and the discussion now seems to endorse this idea), the introduction should also bring this into focus to increase coherence and consistency throughout the text. Potential readers would benefit from it.”*

RESPONSE: We see the Reviewer’s point. However, we were reluctant to reframe the introduction too much in order to maintain an accurate representation of our motivation. However, we also agree that coherence and consistency are important. Thus, as suggested by Reviewer A, we now specify in the introduction that the inhibition tasks mostly tap into task-specific interference-control processes (see p. 5-6): “Overall, this research has led some authors to the conclusion that the tasks used to assess inhibition only assess the ability to reduce the interference arising in that particular task (Rey-Mermet et al., 2018, 2019). In this case, the inhibition tasks mostly tap into interference-control processes, which are highly task specific.

In summary, previous individual-differences research has put forward inhibition as a core interference-control mechanism related to WM. Nevertheless, there is some evidence that establishing inhibition as a valid and reliable construct at the latent-variable level is more difficult than previously thought. This emphasizes that inhibition might not be a general construct, thus mandating us to be very cautious when referring to the concept of “inhibition”.”

Please note that we opted to use the term “task-specific” instead of “mechanism-free”. In our opinion, the term “mechanism-free” might lead readers to conclude that the inhibition tasks involve no interference-control processes at all. However, in order for participants to perform the tasks, we think that some interference-control processes are required. Whether these processes include an active suppression of irrelevant information, a strengthened activation of the correct response, and/or simply the passive decay of the interference from the irrelevant information is still a question of debate and an issue for future research. However, Rey-Mermet et al. (2018, 2019) as well as the present results clearly show that these processes did not generalize across the different inhibition tasks, explaining why we preferred the terminology of “task-specific” interference-control processes.

***A2 “By the way, the new paragraph in the discussion includes the following statement: ‘Thus, although the present study supports the notion that removal is not an inhibitory process…’. Again, I DO NOT think this is true. In fact, it seems quite contradictory to the idea that maybe the used ‘inhibition’ tasks do not measure inhibition.”***

RESPONSE: We changed the sentence as follows (see p. 34): “although the present study supports the notion that removal is not related to the interference-control processes assessed by the inhibition tasks”.

***A3 “Related to this, it is now clear to me that the title of the manuscript should be changed. I cannot see the point of having “inhibition” in it.”***

RESPONSE: We changed the title as follows (see p. 1): “Interference control in working memory: Evidence for discriminant validity between removal and inhibition tasks”. We think that having “inhibition” in the title is important given the paper might be of interest to scholars interested in inhibition.

***A4 “The Stop-Signal Task has been widely used to investigate individual differences (a recent example here*** [***https://www.jneurosci.org/content/38/36/7887***](https://www.jneurosci.org/content/38/36/7887)***). Thus, to justify that you did not pick this task for your study, you better use a different reason.”***

RESPONSE: We thank Reviewer A for pointing out this imprecision. Our point was that the versions of the stop-signal task that minimize the use of strategies have rarely been used in individual-differences research using a CFA and SEM approach, such as the approach used in the present study. This has now been clarified in the manuscript (see p. 36).

***A5 “In response to a (very smart) suggestion by the other reviewer, the authors write: ‘We decided not to mention the retrieval practice paradigm because this task does not belong to the tasks commonly used to assess inhibition in individual-differences’. Well, the authors could want to know that the manuscript already includes mentions to the retrieval practice paradigm (‘… the field will need to specify how inhibition could act on irrelevant/outdated information in WM, and should attempt to build experimental paradigms that are able to disentangle inhibition from other mechanisms of conflict resolution (as has been done with inhibition in episodic memory [73,74]’). 73 and 74 refer to retrieval practice studies. Also, and despite the authors think, the RP (sometimes named ‘retrieval-induced forgetting’) paradigm has been extensively used to explore individual differences in inhibitory control [over episodic memory, of course; have a look at the meta-analytic review by Murayama et al. (2014) here*** [***https://pubmed.ncbi.nlm.nih.gov/25180807/***](https://pubmed.ncbi.nlm.nih.gov/25180807/)***]. Obviously, I totally endorse the original suggestion made by the other reviewer.”***

RESPONSE: We again thank Reviewer A for pointing out another imprecision. Previous research has clearly shown that inhibition in episodic memory – as assessed with the retrieval practice paradigm – differs from the interference-control processes measured in tasks such as the Stroop and flanker tasks (e.g., Friedman & Miyake, 2004; Pettigrew & Martin, 2014). Based on this research, we made a distinction between the interference-control processes measured with paradigms such as the retrieval practice paradigm (which we refer to as “inhibition in episodic memory”) and the interference-control processes measured with the inhibition tasks, such as those used in the present study. Therefore, when we wrote in our previous response that the retrieval practice paradigm “does not belong to the tasks commonly used to assess inhibition in individual-differences research”, we meant that this paradigm does not belong to the tasks typically called “inhibition tasks” (e.g., Stroop flanker, Simon, antisaccade tasks).
